# Do Adolescents Who Meet Physical Activity Recommendations on Weekdays Also Meet Them on Weekends? A Cross-Sectional Study in Colombia

**DOI:** 10.3390/ijerph18030897

**Published:** 2021-01-21

**Authors:** Fernando Galindo-Perdomo, Carmen Peiró-Velert, Alexandra Valencia-Peris

**Affiliations:** 1 Physical Education Program, South Colombian University, Neiva 410001, Colombia; fernando.galindo@usco.edu.co; 2 Department of Teaching of Musical, Visual and Corporal Expression, University of Valencia, 46022 Valencia, Spain; carmen.peiro@uv.es

**Keywords:** gender, organized sport, physical activity recommendations, type of day, active lifestyles

## Abstract

The purpose of this study was to examine whether Colombian adolescents fulfill physical activity (PA) recommendations by type of day depending on several variables. A cross-sectional study was carried out on a sample of 2624 adolescents from Neiva (Colombia) using the Seven Day PA Recall and the Family Affluence Scale II self-reported questionnaires. Statistical analyses were performed to find any differences by gender, socioeconomic status, PA recommendations accomplishment, participation in organized and non-organized PA, parents’ involvement in sport, and adolescents’ academic achievement, and to determine the probability of complying with the PA guidelines. Only 12.3% of the participants met the PA recommendations on weekdays and weekends. Girls maintained their (in)active patterns during the week more than boys (84.4% vs. 70.7%, respectively), while more boys than girls changed them according to the type of day (25.3% vs. 19.6%). Participation in organized sports was the predictor variable with the highest probability of both boys and girls complying with the PA recommendations on weekdays. Non-organized activities were more likely to make girls more active on weekends. In conclusion, a wider offer of organized and non-competitive PA over the weekend and broader sports schedules should be provided to youth in Neiva.

## 1. Introduction

Research indicates that regular physical activity (PA) has beneficial health effects in childhood and adolescence [1,2]. Children and adolescents are recommended to perform at least 60 min per day of moderate-to-vigorous physical activity (MVPA) to reduce the risk of cardiovascular disease, to improve body composition and cardiorespiratory and musculoskeletal fitness, and promote normal growth and development [3,4,5].

Nevertheless, international research reveals a negative trend in adolescents’ PA participation in Western societies and accomplishment the World Health Organization (WHO) PA recommendations [4]. This trend can be found in studies conducted in the USA and Canada [6,7], Europe [8,9,10,11], Korea [12], and in a multinational study [13,14]. They consistently showed that a higher percentage of those who meet the recommendations are males, younger students and those with higher socioeconomic status (SES). This trend is also evident in some Colombian studies conducted on children and adolescent populations [15,16,17,18]. Two of these descriptive studies reported that 26% of the participants met the recommendation for engaging in MVPA for 60 min per day [16,17]. Another study on schoolchildren in the city of Bogotá indicated that 36.9% accomplished the PA recommendations [18]. Piñeros and Pardo’s work with 13-to-15-year-old adolescents from five Colombian cities revealed that 15% engaged in PA at least 60 min/day during the entire week [19]. All the studies mentioned found a gender difference, where boys were more active than girls.

Regarding the type of day (weekday/weekend), some international studies showed that children and adolescents spend more time in moderate PA, vigorous PA, or MVPA on weekdays than on weekends [20,21]. Considering that children and adolescents have greater academic commitments and homework on weekdays, it would be logical to think that they would better comply with the PA recommendations on weekends given that they have greater options to choose how to spend their time, but this is not the case. The studies in this line of research agree that children and adolescents better comply with the recommendations during weekdays than on weekends [22,23] and also indicate that boys and youngsters who receive family support largely comply with the PA recommendations on both weekdays and weekends, unlike their counterparts. It has also been shown that on weekends, adolescents devote more time to sedentary behavior and less to PA [24,25]. In Colombia, to date, only one study has examined the time spent on MVPA with respect to the type of day. This study was part of a multinational cross-sectional survey conducted in 12 countries investigating schoolchildren between 9 and 11 years old and showed that they spent an average of 70.8 min per day on MVPA on weekdays and 62 min per day on weekends [23].

It is therefore of interest to study other factors that make children and adolescents largely comply with the recommendations during the week and not on weekends, and vice versa. Some studies have shown the importance of parents’ support for children and adolescents’ active behavior. Parents who are physically active, those who are aware of the benefits of PA, those who provide support and accompany their children, and those who provide motivational and economic support are essential for children and adolescents to meet the PA recommendations [26,27]. Participation in organized and non-organized sports is also associated with high levels of PA and with a higher proportion of children and adolescents who fulfill the recommendations [28,29,30]. In Switzerland, boys who participate in a sports club and have physically active parents are associated with higher percentages of compliance with PA recommendations on weekdays [8]. British children who have family encouragement and carry out family activities present higher levels of MVPA over the weekend [26]. Other studies have also shown that complying with the MVPA recommendations is associated with better academic performance [31,32]. 

In line with this study, it seems necessary to guide future lines of research toward PA engagement on weekends [22,33] and to determine the factors associated with young people’s accomplishment of PA recommendations and active behavior throughout the week [34,35,36]. This is particularly important in Colombia, where there is at present very little data available on these factors. This study thus had a twofold aim: first, to examine whether Colombian adolescents behave differently regarding PA depending on the type of day (weekdays and weekends) and gender. Second, to focus on those who change their active profile by type of day in order to determine the likelihood of being active only on weekdays or only on weekends with regard to the different variables involved in PA participation.

## 2. Materials and Methods 

### 2.1. Sample

The participants in this cross-sectional study were 2624 adolescents who belonged to 8 schools (4 state and 4 private) in the city of Neiva (Colombia), aged from 11 to 18 years (M = 13.7 years, SD = 1.4 years). Proportional stratified sampling was performed according to different variables: type of school (state or private), school grade (sixth to ninth), and gender. The characteristics of the sample are shown in Table 1.

### 2.2. Instruments and Ethical Considerations

Several instruments were employed to collect the data of the variables in the study, such as PA, SES, type of day of the week, and academic performance. The Seven-Day Physical Activity Recall (7-Day PAR) [37], which has been proven reliable and valid [38], was used to determine the time students were involved in PA on weekdays and weekends, as well as the intensity of the PA (i.e., moderate, vigorous, and MVPA), during the last 7 days. However, as it has recently shown low validity regarding vigorous and moderate PA, the administration protocol was improved to obtain reliable data in these PA categories [39]. The Spanish version adapted to the school population was administered as in previous studies [40]. Participants were classified depending on whether they met the PA guidelines (60 min per day of MVPA) [4] on weekdays and weekends.

The Family Affluence Scale II [41] was used to determine the participants’ SES since this scale reflects the family’s material resources and the specific purchasing power that is allowed by their family income (the items include family cars, computers, number of vacation trips, child’s bedroom). Regarding the scoring system, the responses of each of the four items were added, achieving a number between 0 and 9 points, with 0 being the lowest purchasing power and 9 being the highest. Consistent with the international protocol, scores ≤2 indicated a low SES, from 3 to 5 indicated a medium SES, and scores ≥6 indicated a high SES [42].

The students were also asked about their academic achievement by responding to the number of subjects they failed or passed in the previous academic period (i.e., fail one or more subjects or pass all subjects). They also declared any participation in any organized and/or non-organized sport in after-school sports and whether their parents were involved in any sport.

The work was carried out in the second half of 2017. All the subjects gave their informed consent for inclusion before they participated in the study, which was conducted in accordance with the Declaration of Helsinki. The protocol was approved by the Ethics Committee of the University of Valencia (H1488452044602) and authorized by the Neiva Municipal Education Secretariat and the participating schools. Informed consent forms were completed by the parents and tutors of each of the participants, who were guaranteed full anonymity.

### 2.3. Data Analysis

After coding, cleaning, and grouping of the data, different statistical analyses were carried out using SPPS v.24.0 software (IBM SPSS Statistics for Windows, Armonk, NY: IBMCorp. USA). It was found that the data did not meet the assumptions of normality (using the Kolmogorov–Smirnov test) and homoscedasticity (using Levene’s test). To avoid performing non-parametric tests in the analyses, the variables were transformed using the square root.

First, *t*-tests and chi-square tests were carried out on the continuous and categorical variables, respectively, to determine the differences between boys and girls, SES, accomplishment of the PA recommendations on weekdays and weekends, engagement in sports (sports clubs), participation in non-organized PA, parents’ involvement in sport, and adolescents’ academic achievement. Binomial logistic regression was then carried out to determine the likelihood of complying with PA recommendations only on weekdays, or only on weekends, for the entire sample and by gender according to SES, participation in sports clubs, participation in non-organized PA, having active parents, and academic achievement. The odds ratios were established with 95% confidence intervals (CIs).

## 3. Results

The overall characteristics of weekday and weekend (non)compliant participants, stratified by gender, are summarized in Table 2. In general, most Colombian adolescents (78%) from Neiva maintained their (in)active behavior, regardless of the type of day. A total of 12.3% of the students met the PA guidelines both on weekdays and weekends, and 65.6% did not accomplish the PA recommendations on any type of day. However, 22% changed their behavior, either being physically active only on weekdays (11.2%) or only on weekends (10.8%).

Regarding differences by gender, girls maintained their behavior throughout the week more than boys (84.4% vs. 70.7%, respectively) and boys changed their weekday/weekend (in)active patterns more than girls (25.3% vs. 19.6%). There were more active boys both on weekdays and weekends than active girls (*χ*^2^ = 69,786, *p* < 0.05, Cramer’s *V* = 0.163) and more inactive girls than inactive boys on both types of day (*χ*^2^ = 174,842, *p* < 0.05, Cramer’s *V* = 0.258). Taking into account the behavior changes by type of day, there were also more active boys on weekdays who were inactive on weekends than girls (*χ*^2^ = 62.494, *p* < 0.05, Cramer’s *V* = 0.154), but more active girls on weekends who were inactive on weekdays than boys (*χ*^2^ = 11.130, *p* < 0.05, Cramer’s *V* = 0.065). Concerning the determinants of PA participation, boys participated more in sports clubs (*χ*^2^ = 70.712, *p* < 0.05, Cramer’s *V* = 0.164) and in non-organized PA (*χ*^2^ = 101.232, *p* < 0.05, Cramer’s *V* = 0.196), reported worse academic achievement (one or more fails) (*χ*^2^ = 43.645, *p* < 0.05, Cramer’s *V* = 0.129), and their parents engaged more in PA (*χ*^2^ = 50.370, *p* < 0.05, Cramer’s *V* = 0.150) than girls.

Two binary logistic regression analyses were conducted to determine the likelihood of being active only on weekdays or only on weekends, and vice versa (Table 3), according to SES, PA and sports participation, active parents, and academic achievement. A model was built for the whole sample and the analysis was then repeated according to the adolescents’ gender. The analysis that included all the adolescents showed that the predictor variables in relation to being inactive on weekends and active on weekdays were (in order of likelihood): participating in sports clubs (6.5 times higher) and in non-organized PA (1.7 times higher). The SES, having active parents, and academic achievement variables did not reduce or increase the likelihood of being active during weekdays. Comparing the prediction model for boys and girls, the results revealed that engagement in sports clubs’ activities predicted both genders to be active, while non-organized PA did not affect girls’ meeting the PA guidelines on weekdays.

On the other hand, the determinants of being active only on weekends but not on weekdays were (in order of likelihood): participating in non-organized PA (3.3 times higher) and in sports clubs (1.4 times higher). Comparing the prediction model for boys and girls, the results indicated that non-organized PA (2.45 times higher) and sports clubs, to a lesser extent, predicted girls’ meeting the PA recommendations on weekends. For boys, sports clubs did not affect being active on weekends and inactive on weekdays. However, non-organized PA (2.97 times higher) and having parents engaged in PA, to a lesser extent, predicted being active on the weekend. Once again, SES and academic achievement did not reduce or increase the likelihood of being active during weekends.

## 4. Discussion

This is the first study to analyze the profiles of Colombian adolescents that considers whether PA recommendations were met according to the type of day and several sociodemographic variables. It first examined the prevalence of the overall (non)compliant participants regarding their time involved in PA on weekdays and weekends, as well as the prevalence stratified by gender. Consistent with previous research in other countries, the results revealed that adolescents in Neiva were more active on weekdays than on weekends [9,22,25]. It also found that although the adolescents showed similar percentages regarding meeting the PA recommendations of 60 min of MVPA per day on weekdays (but not on weekends) (11.2%), on weekends (but not on weekdays) (10.8%), or both types of day (12.3%), they differed by gender. Hence, while Colombian boys were more active on weekdays (and inactive on weekends) than girls and more active on both types of day, female adolescents were more active on weekends (and inactive on weekdays) than boys. The lower girls’ involvement in PA on weekdays than boys concurs with a previous study conducted in Chile [43]; however, it contrasts with a study on British students [21], which showed that girls were more active on weekdays than on weekends. In the Colombian context, and especially in the city of Neiva, this situation can be explained by a number of reasons. According to previous national research [16,17], Colombian girls spend more time than boys on sedentary behavior, such as social networking (particularly via cell phones), and engage more in low-energy activities rather than MVPA. Likewise, other international studies agree that girls are more committed to academic duties on weekdays, and these duties could compete with vigorous activities for their limited leisure time [44,45]. Another reason that may explain why female adolescents spend less time on PA on weekdays is related to a major concern of the Colombian Government’s security policy, namely, homicide [46], with the Department of Huila ending 2019 with 243 cases (the fifth most regarding violent deaths) and Neiva being one of the cities with the highest rates [47]. This perception of insecurity in their community environment may make girls’ parents reluctant to allow their daughters to attend outdoor sports facilities on weekdays. While most of the parents can accompany them on weekends, they cannot do so on weekdays due to work duties; therefore, girls would have to go alone and be exposed to possible violent assaults. According to the 2019 Forensis Report [47], 65.14% of the adolescents assaulted were girls, and most cases occurred on weekdays between 15:00 and 23:52. These sociocultural situations require political intervention to ensure female adolescents can find secure sports scenarios to safely engage in PA.

The second contribution of the present study was related to its aim of adding to the knowledge of the role of several factors in the likelihood of students complying with PA recommendations during the week, but failing to meet them on the weekend, and vice versa. First of all, it should be noted that SES did not seem to be related to the accomplishment of PA guidelines in adolescents in Neiva, either on weekdays or weekends. Although SES is known to influence PA, there are inconsistencies that support the fact that this relationship is universal [42]. Some studies have not found associations between SES and PA [48], or have even demonstrated that adolescents from higher SES families meet the PA guidelines to a lesser extent [49]. In this context, the availability of outdoor facilities can play an important role for adolescents from lower SES families or who come from a deprived neighborhood. The results showed the importance of participation in both sports clubs and non-organized PA as predictors of Colombian adolescents being active on weekdays and inactive on weekends. On the other hand, it revealed the significance of sports facilities in the accomplishment of PA recommendations for boys and, to a lesser extent, for girls. These findings are in agreement with a Spanish study on adolescents that found that 49.5% of boys and 32.1% of girls who were involved in organized sports after school during the week complied with the recommendation of 60 min/day of PA [50]. Furthermore, in a sample of Swiss children and adolescents, to be a male participant and engage in sports clubs were associated with the accomplishment of PA recommendations on weekdays [8]. In the same vein, a higher percentage of Portuguese boys from 10 to 18 years old who participated in sports clubs fulfilled the PA recommendations (28.3%) than girls (7.7%) [51]. Overall, sports clubs, as the key institutions in organizing sports activities, seem to stand out as an enabling environment for fostering organized PA and helping to facilitate compliance with PA recommendations [52,53,54]. Nevertheless, a particular issue in our study was linked to the timetables on offer in these organizations. In the city of Neiva, sports clubs, school sports, or sports leagues usually offer training schedules on weekdays, while official league matches or championship games are held on the weekend. This programming of the sports schedules provides a limited offer of organized and non-competitive PA to children and adolescents in Neiva over the weekend and does not enhance the likelihood of young people’s compliance with PA recommendations.

On the other hand, along with the importance of sports clubs and their offer of organized non-competitive PA on weekdays, the findings revealed that non-organized PA has the highest probability of meeting the PA recommendations on the weekend. The reason for this may be that Neiva adolescents have few or no duties or sports training on weekends; therefore, they can spend more free time in non-organized PA. As far as gender is concerned, our results showed that girls were more likely than boys to comply with the recommendations by performing non-organized PA on weekends. However, this finding does not match with prior research. In Norway, girls from 13 to 18 years old spent less time each week in non-organized PA than boys [30]. In Canada, adolescent girls aged 12 to 16 years participated less frequently in non-organized activities than boys, both in and out of school [28]. Likewise, boys in Ontario (Canada) aged 11 to 20 years were more likely to engage in outdoor activities every day of the week, and this was significantly associated with meeting PA recommendations [29].

Parental role modeling has been found to be crucial in developing healthy and active lifestyles in adolescents [55,56], with boys perceiving their families as playing an active role in supporting their engagement in PA more than girls do. Our findings support the existing literature on this issue and show that physically active parents (64.6%) emerged as a predictor for boys complying with PA recommendations, particularly on weekends, together with their participation in non-organized sport. Some factors may influence these findings, such as the offer of urban PA programs and parents’ availability to share participation in these programs with their sons. Previous Colombian studies found that the users of the Ciclovía recreational program had a higher prevalence of meeting PA recommendations than non-users [57,58]. In the city of Neiva, this program closes the streets to motor vehicles on weekends, which means they are open for leisure activities. Recreational programs of this type should be fostered since they facilitate family PA participation, particularly on weekends when parents do not usually work and are more likely to accompany their children in exercise. Evidence from other studies has also shown associations between parents’ engagement in PA and their children’s engagement by type of day. A study on Portuguese adolescents aged 12 to 18 years whose father or mother engaged in PA were more active and had a greater chance (4–6 times a week) of performing organized and non-organized PA [35]. In England, 9- and 10-year-olds who received high levels of family encouragement, family social support, and whose parents practiced PA were more physically active on weekends than those children who did not [26]. In Canada, Vander Ploeg et al. [36] found that parents’ involvement in PA, their beliefs, and support for PA were associated with children being more physically active on school days and on weekends. A study conducted in the Czech Republic indicated a relationship between parents’ and children’s engagement in PA, especially on the weekend [59]. Furthermore, in the Czech Republic, PA was measured by counting the number of steps taken by children (aged 5 to 12 years old) and their parents in a typical week. Positive associations were found between the parents’ and children’s step counts, both during the week and on weekends, and were higher on the weekend [27]. In the United States of America, a positive relationship was found between mothers’ and children’s MVPA. This relationship was stronger in the evenings and on weekends, when mothers and children could share time together [60].

Evidence from some systematic reviews on relationships and the effects of PA and academic achievement show that although these relationships indicate positive increases in academic achievement in physically active students, the causal roles of numerous PA elements in academic achievement remain to be explored [61,62]. In particular, the Position Stand written by Donnelly et al. suggests that PA may have a neutral effect on academic achievement [62]. Consistent with this finding, in the present study, academic achievement did not affect the likelihood of boys or girls being active. There is a peculiarity in Neivan educational policies concerning sports programs, in which only students achieving good results are allowed to participate in interschool sports tournaments or competitions. This is not the case for involvement in sports clubs, where any adolescent can participate regardless of their academic record. Since we did not distinguish between engagement in school sports programs and sports clubs in the present study, their contextual differences in participation may help to explain this finding. Future studies conducted in the Huila department should therefore make this distinction when studying the relationship between PA and academic achievement. However, in line with other studies that found positive associations between PA and academic achievement, the National policy initiatives in Colombia, such as “Supérate con el deporte” (Excel yourself with sport), “Currículo para la excelencia académica y la formación integral 40 × 40” (Curriculum for academic excellence and comprehensive education 40 × 40), or “Muévete escolar” (Let’s move at school), may help to increase PA levels and improve the learning environments and academic achievement [17]. 

The present findings may contribute to expanding future lines of research on Colombian children and adolescents’ compliance with PA recommendations by the type of day. However, as it was a cross-sectional study, no causal inferences can be made between adolescents’ accomplishment of PA recommendations, the type of day, and sociodemographic variables (participation in sports clubs, non-organized PA, and active parents). Longitudinal studies should therefore be carried out to examine the effects of these variables over time. Another limitation is related to the fact that the PA measure was self-reported. Due to previous evidence that found that subjective methods of data collection may overestimate or underestimate the information reported by teenagers [63], some concern arises as to the reliability of the data. However, the questionnaire used was previously proven to be reliable and valid [38], it has been used in several international studies [37,40], and errors were minimized by employing standardized protocols and guidelines [39]. It would thus be interesting to consider other variables in the analysis, such as media screen usage, sedentary behavior, and weight status to determine whether overweight or obese children behave similarly. In this regard, identifying young Colombians’ free-time active and sedentary behavior and relating them to the accomplishment of the PA recommendations could help to develop public policies that improve healthy and active lifestyles.

## 5. Conclusions

To conclude, our results showed that adolescent schoolchildren were more active and met the recommendations in a greater proportion during the week than on weekends. Taking into account gender, a higher proportion of boys met the PA recommendations on weekdays and weekends than girls. Likewise, organized sport emerged as an important issue in adolescents’ compliance with the PA recommendations on weekdays. Therefore, this highlights the importance of the PA on offer from sports clubs, training schools, and sports leagues in keeping adolescents physically active. It is evident that by getting involved in unorganized sport on weekends, adolescents are more likely to comply with the PA recommendations. In this regard, the availability of outdoor sports facilities becomes crucial for enabling children and adolescents to safely engage in physical activities.

## Figures and Tables

**Table 1:** Sociodemographic characteristics of the sample.

Variables	Frequency	Percentage
Gender		
Female	1395	53.0
Male	1229	47.0
Participation in sports clubs		
Yes	1021	38.9
No	1603	61.1
Participation in non-organized PA		
Yes	1496	57.0
No	1128	43.0
Parents who play sports		
Yes	1277	48.7
No	1347	51.3
Academic achievement		
One or more fails	1225	46.7
No fails	1399	53.3

PA: physical activity.

**Table 2:** Characteristics of weekday and weekend (non)compliant participants with physical activity recommendations, overall and by gender.

Characteristics	All (*n* = 2624)	Girls (*n* = 1395)	Boys (*n* = 1229)	*p*
Activity profile by type of day				
Active weekdays–active weekends (%)	324 (12.3)	102 (7.3)	222 (18.1)	<0.001
Active weekdays–inactive weekends (%)	295 (11.2)	93 (6.7)	202 (16.4)	<0.001
Inactive weekdays–active weekends (%)	283 (10.8)	159 (12.9)	124 (8.9)	<0.001
Inactive weekdays–inactive weekends (%)	1722 (65.6)	1076 (77.1)	646 (52.6)	<0.001
SES (%)				
Low	255 (9.7)	133 (9.5)	122 (9.9)	0.112
Middle	1492 (56.9)	819 (58.7)	673 (54.8)	
High	877 (33.4)	443 (31.8)	434 (35.3)	
Type of participation				
Participation in sports clubs (%)	1021 (38.9)	438 (31.4)	583 (47.4)	<0.001
Participation in non-organized PA (%)	1496 (57)	668 (47.9)	828 (67.4)	<0.001
Active parent (%)	1277 (56.8)	595 (49.8)	682 (64.6)	<0.001
Academic achievement (%)				
One or more fails	1225 (46.7)	567 (40.6)	658 (53.5)	<0.001
No fails	1399 (53.3)	828 (59.4)	571 (46.5)	

SES: socioeconomic status; PA: physical activity.

**Table 3:** Binary logistic regression to predict whether the PA guidelines were met on weekdays and the weekend for the entire sample and by gender.

Predictors	Active Only on Weekdays						Active Only on Weekends					
	All (*n* = 295)		Girls (*n* = 93)		Boys (*n* = 202)		All (*n* = 283)		Girls (*n* = 159)		Boys (*n* = 124)	
	OR	95% CI	OR	95% CI	OR	95% CI	OR	95% CI	OR	95% CI	OR	95% CI
SES	1.07	0.91–1.27	0.88	0.64–1.21	1.05	0.86–1.28	1.08	0.92–1.26	0.93	0.71–1.22	1.10	0.92–1.34
Sports clubs												
No	1 (ref.)		1 (ref.)		1 (ref.)		1 (ref.)		1 (ref.)		1 (ref.)	
Yes	**6.56**	**4.83–8.90**	**4.89**	**3.03–7.89**	**6.98**	**4.63–10.52**	**1.41**	**1.06–1.89**	**1.56**	**1.01–2.40**	1.26	0.85–1.86
Non-organized PA												
No	1 (ref.)		1 (ref.)		1 (ref.)		1 (ref.)		1 (ref.)		1 (ref.)	
Yes	**1.72**	**1.28–2.30**	1.32	0.83–2.10	**1.63**	**1.10–2.43**	**3.37**	**2.45–4.65**	**3.42**	**2.19–5.34**	**2.97**	**1.84–4.80**
Active parent												
No	1 (ref.)		1 (ref.)		1 (ref.)		1 (ref.)		1 (ref.)		1 (ref.)	
Yes	1.14	0.84–1.53	1.26	0.78–2.05	0.99	0.66–1.48	1.25	0.93–1.68	0.92	0.60–1.40	**1.61**	**1.05–2.47**
Academic achievement												
One or more fails	1 (ref.)		1 (ref.)		1 (ref.)		1 (ref.)		1 (ref.)		1 (ref.)	
No fails	0.91	0.69–1.21	1.04	0.65–1.65	0.99	0.68–1.44	0.92	0.70–1.22	1.15	0.76–1.75	0.81	0.55–1.21

OR: odds ratio; CI: confidence interval; SES: socioeconomic status; PA: physical activity; ref.: Reference. Significant effects are shown in bold (*p* < 0.05).

## Data Availability

The data presented in this study are available on request from the corresponding author. The data are not publicly available due to ethical reasons.
